# Translation to German and linguistic validation of the Rapid Assessment of Physical Activity (RAPA) questionnaire

**DOI:** 10.1186/s41687-023-00649-w

**Published:** 2023-10-31

**Authors:** Stefan Tino Kulnik, Johanna Gutenberg, Kathrin Mühlhauser, Tari Topolski, Rik Crutzen

**Affiliations:** 1 Ludwig Boltzmann Institute for Digital Health and Prevention, Lindhofstrasse 22, 5020 Salzburg, Austria; 2 Department of Health Promotion, Care and Public Health Research Institute (CAPHRI), Maastricht University, Maastricht, Netherlands; 3 Department of Health Services, University of Washington, Seattle, USA

**Keywords:** Cognitive debriefing, Linguistic validation, Patient-reported, Qualitative, Self-reported, Translation

## Abstract

**Purpose:**

To produce a culturally adapted translation of the Rapid Assessment of Physical Activity (RAPA) questionnaire for German speaking Austrians and to conduct a linguistic validation of the new language version.

**Methods:**

The original English RAPA questionnaire was translated into German for Austria and underwent an independent forward and back translation, followed by cognitive debriefing interviews with older adults aged 55 to 78 years with and without health conditions (*n* = 13), for linguistic validation.

**Results:**

Several distinct choices were made in the translation of the RAPA questionnaire to German, including the use of colloquial terms for ‘physical activity’ and ‘intensity’; and the decision to keep to the original examples and images of different physical activities for illustrating the intensity levels (light, moderate, vigorous) of physical activity. In cognitive debriefing, interviewees commented that some example activities for the respective intensity levels could – depending on the individual – also represent a higher or lower intensity level; and that the wording of RAPA items 4 and 5, which describe the category ‘under-active regular’ aerobic activity, was difficult to understand. Both issues were addressed and resolved through minor iterative modifications made during the cognitive debriefing process.

**Conclusions:**

A new version of the RAPA questionnaire in German for Austria has been produced by forward and back translation and linguistic validation. The questionnaire may now undergo psychometric evaluation.

## Introduction

Regular physical activity constitutes one of the most important human health behaviours that is applicable to all ages along the life span, and across healthy individuals and groups with chronic health conditions and disabilities alike. The current World Health Organization (WHO) recommendations for adults (including older adults, individuals living with chronic conditions and individuals living with disabilities) call for 150–300 weekly minutes of moderate-intensity or 75–150 weekly minutes of vigorous-intensity aerobic physical activity, or a combination of both [1, 2]. In order to promote physical activity in routine healthcare contexts, it is helpful to capture individual levels of physical (in)activity in an affordable and easy way, for example using self-report tools.

The Rapid Assessment of Physical Activity (RAPA) questionnaire was developed to provide an easily administered and interpreted means of assessing levels of physical activity among older adults [3]. It is a self-reported questionnaire consisting of nine statements with binary response options (yes/no). The first seven statements describe a certain frequency and intensity of weekly physical activity (e.g., ‘I do some light physical activity every week’) in increasing order of frequency and intensity. The eighth and ninth statements describe a certain frequency of physical activity for muscle strengthening and for flexibility, respectively. The questionnaire is prefaced with accessible explanations of the term ‘physical activities’ and the difference between light, moderate and vigorous intensity levels, including images of example activities for each intensity level. In the scoring, respondents are categorised to one of five activity levels according to their highest self-rated frequency and intensity of weekly physical activity: sedentary, under-active, under-active regular – light activity, under-active regular, or active. In its development of the original English version, the RAPA was compared to several regularly used physical activity assessments among older adults in the USA, including the Community Health Activities Model Program for Seniors of regular physical activity [3].

A recent scientific statement of the American Heart Association included a comparative evaluation of 14 short self-report tools for assessing physical activity in healthcare settings [4]. In this evaluation, the RAPA was rated highest by a composite score of content validity, coverage of aerobic and muscle strength components of physical activity guidelines, test–retest reliability and clinical feasibility [4]. The RAPA could therefore prove a useful tool for healthcare professionals to support the promotion of physical activity in Austria and other German-speaking countries, but a validated German version of the RAPA has not been developed to date. This article describes the process and outcome of the translation and linguistic validation of the RAPA to German for Austria.

## Methods

The original English RAPA questionnaire was translated into German for Austria and underwent an independent forward and back translation, followed by cognitive debriefing interviews for linguistic validation. The specification of Austrian German is made to differentiate from German for Switzerland or German for Germany, as there are differences in grammar and vocabulary across German-speaking countries and populations [5].

### Translation from the original English source version

A translation of the RAPA questionnaire to German for Austria was developed following the recommended stepwise method of independent forward and back translation [6, 7]. Two forward translations of the original English language version (source questionnaire) [8] to German (Austrian) were prepared independently by two translators (STK, JG) who are both native German speakers with experience in physical activity research, public health, and health promotion in clinical settings. STK is native to Austria, and JG is native to Germany and has lived in Austria for several years. STK and JG then compared and discussed the two translations and reconciled them into one version. A back translation of the reconciliated forward version to English was conducted by an independent translator and native English speaker without prior reading of the English source version. An independent consultant (RC) then compared the back translation with the source questionnaire to ensure faithful translation.

### Cognitive debriefing

For cognitive debriefing, we recruited 13 older adults who were representative of the intended target group for the RAPA questionnaire. Participants aged 55 years and older who were proficient German speakers and native to Austria were invited via the research institute’s patient and public involvement database. Recruitment was purposive, aiming for representation of men and women, and including healthy participants as well as individuals with relevant medical conditions such as heart disease.

Cognitive debriefing interviews [9] were conducted individually by experienced qualitative researchers (STK, JG, KM) at quiet and private locations conducive to qualitative interviewing. All interviews were held in German and audio-recorded. Each participant first self-completed the RAPA questionnaire (pen and paper). The researcher noted the time it took the participant to complete the questionnaire. To assess the participant’s understanding and difficulty of completing the questionnaire, the researcher then asked several probing questions and took concurrent notes. The following probing questions were adapted from Brod et al. [9]:

- Can you describe in your own words what the questionnaire is about?
- Please tell me what you generally thought about the questionnaire?
- Did the explanation of different levels of intensity/effort of physical activity make sense to you?
- What did these questions mean to you?
- Were the questions worded in a way that made sense to you?
- Were the questions in any way offensive or objectionable to you?
- Were the instructions and the formatting/layout clear?
- Did the response options make sense to you?
- How did you select your response?
- When you completed the questionnaire, do you think you were able to accurately judge your physical activity according to the given categories?
- Is there anything else you would like to comment on regarding this questionnaire?

### Analysis

Interviews were conducted in blocks of up to three consecutive interviews. Each block of interviews was conducted with different participants. After each block of interviews, an interim analysis was conducted by STK who listened back to interview recordings and reviewed interviewers’ notes. STK tabulated participants’ comments in relation to (a) questionnaire design (layout, font size, spacing on the page), (b) offensive or questionable content, (c) explanation of physical activity and intensity, (d) wording of questions and (e) response options.

The questionnaire was revised as required after each block of interviews, until feedback from five consecutive interviewees suggested that the questionnaire was appropriate with regard to comprehensibility and acceptability of content and formatting/layout. Interview findings and revisions to the questionnaire were iteratively discussed and agreed upon among the authors, including the developer of the English source version (TT).

## Results

### Translation

Notable discussion and decision points in the reconciliation between the two independent forward translations of the RAPA questionnaire were:

- The use of ‘körperliche Bewegung’ (‘physical movement’) in the introduction was considered preferable over the literal translation ‘körperliche Aktivität’ (‘physical activity’), and the use of ‘sich bewegen’ (‘to move’) in items 2–7 was considered preferable over the literal translation ‘körperlich aktiv sein’ (‘to be physically active’), because they better reflect everyday use of language in German (Austrian). Similarly, the use of ‘Anstrengung’ (‘effort’/’exertion’) in the introduction, description of intensity of different activities, and wording of items 2–7 was considered preferable over the literal translation ‘Intensität’ (‘intensity’) because it is more reflective of lay language and places more emphasis on personal experience of exercise intensity.
- In the introduction, the example activities and images were reviewed, and it was discussed whether these were representative for the local population or whether alternative or additional activities and images should be used. Of note, RAPA translations to other languages have introduced alternative or additional example activities and images, such as dancing or playing football in the Mexican Spanish version [10]. Upon reflection, the example activities and images shown in the source questionnaire were considered appropriately representative for the German (Austrian) context.
- In item 8, the example ‘calisthenics’ was not translated because it was considered a rather specific and unfamiliar term. Instead, the example of ‘Hantel- und Krafttraining’ (‘free weights and strength training’) was chosen for the translation.
- In the scoring instructions, the terminology for physical activity categories was aligned with terminology from the Austrian physical activity recommendations [11].

Comparing the original source questionnaire and the back translation, several differences in wording were noted; however, these expressed the same meaning. For example, the phrase ‘An assessment of level and intensity of physical activity’ in the original version became ‘Questionnaire regarding the extent/degree…’ in the back translation; and '…increase your heart rate above its resting rate…' became '…heart pumps faster than lying down or sitting calmly…'. Overall, the reconciled translation of the RAPA questionnaire to German for Austria was considered adequate for linguistic validation through cognitive debriefing.

### Cognitive debriefing

Thirteen older adults (5 women, 8 men) with median age 66 years (range 55 to 78 years) took part in cognitive interviews. Five interviewees were representative of the general (healthy) population, and eight had chronic medical conditions which included cardiac (*n* = 5), neurological (*n* = 1), orthopaedic (*n* = 1), and respiratory (*n* = 1) conditions. Interviewees’ aerobic physical activity categories according to the RAPA represented the range from ‘under-active’ to ‘active’. Average time for completing the RAPA questionnaire was 3 min (range 1.5–5.5 min; Table 1).

**Table 1:** Overview of responses from cognitive debriefing interviews

RAPA version	Version 0.1	Version 0.1	Version 0.1	Version 0.2	Version 0.2	Version 0.2	Version 0.3
Interview number	1	2	3	4	5	6	7
Participant characteristics	Female, age 59 years General population RAPA activity level: active RAPA Score: 6	Female, age 66 years General population RAPA activity level: active RAPA Score: 6	Male, age 60 years General population RAPA activity level: active RAPA Score: 7	Female, age 55 years Orthopedic rehabilitation RAPA activity level: active RAPA score: 10	Male, age 65 years Cardiac rehabilitation RAPA activity level: active RAPA score: 10	Male, age 69 years Cardiac rehabilitation RAPA activity level: under-active regular – light activities RAPA score: 3	Male, age 66 years Cardiac patient RAPA activity level: active RAPA score: 10
Time to complete the RAPA questionnaire	2 min 20 s	2 min	1 min 45 s	2 min 20 s	1 min 30 s	2 min	5 min
Design (layout, font size, spacing on the page)	Design is appropriate	Design is clear and fits well	Design is appropriate	Design is appropriate	Design is clear and it is easy to read (font size)	Design is appropriate	Appropriate
Offensive or questionable content	None, all questions are worded politely	None	None	None	None	None	None
Explanation of physical activity and intensity	Sometimes the examples may not be suitable, for example strength training as well as aerobics class can also be vigorous activities, because the content is not clearly defined	Understandable	The participant found the categories do not apply to his physical activity, because he is regularly doing hard construction work, so it was difficult for him to select the correct answer; maybe the intensity categories and example activities are not suitable for a hard-working person	Understandable, easy to read, even without glasses	Understandable, appropriate graphics	The given examples were easy to understand and graphics were helpful	Helpful to understand levels of exertion in relation to heart rate; the examples could include an image of gardening, which could be a moderate intensity activity; questions the image of an old man for ‘walking at a slow pace’
Wording of the questions	Time specifications in items 4, 5 and 6 are incomprehensible; she had to read these items repeatedly to understand their meaning	Content is meaningful but items 4 and 5 caused some confusion	Content is meaningful and easy to understand	Items 4 and 5 are difficult to grasp, but overall, the content is meaningful	Content is meaningful and easy to go through	Content for items 1–3 is meaningful and easy to understand; items 4–5 were too long and harder to understand (but not impossible); items 6–7 and the items on strength and flexibility were easy to understand	Answered items 4 and 5 incorrectly upon first completion, had to read repeatedly to understand the meaning; suggests to split items 4 and 5 into two items each, asking about each level of activity separately
Response options	Yes and no answer options are appropriate in relation to the questions	Sometimes difficult to choose between yes and no, but then considered for herself whether it is more likely to be yes or no and ticked accordingly; in general, she prefers to define answers herself	Yes and no answer options are appropriate in relation to the questions	Yes and no answer options are appropriate in relation to the questions	Yes and no answer options are appropriate in relation to the questions	Yes and no answer options are appropriate in relation to the questions	Clear, able to relate all response options to himself

RAPA version	Version 0.3	Version 0.4	Version 0.4	Version 0.4	Version 0.4	Version 0.4
Interview number	8	9	10	11	12	13
Participant characteristics	Female, age 71 years General population RAPA activity level: active RAPA Score: 10	Female, 73 years General population RAPA activity level: active RAPA score: 6	Male, 78 years old Neurologic patient RAPA activity level: under-active regular RAPA score: 8	Male, age 56 years Cardiac rehabilitation RAPA activity level: active RAPA score: 6	Male, age 56 years Post-COVID-19 rehabilitation RAPA activity level: active RAPA score: 6	Male, age 73 years Cardiac patient RAPA activity level: under-active RAPA score: 2
Time to complete the RAPA questionnaire	5 min 20 s	2 min 10 s	5 min 30 s	2 min 30 s	2 min 30 s	4 min 20 s
Design (layout, font size, spacing on the page)	Good font size, appropriate layout	Appropriate	Good	Good	Good	Good, font size appropriate for older people
Offensive or questionable content	None	None	None	None	None	None
Explanation of physical activity and intensity	Understandable, but also the level of exertion experienced with any of the example activities depends on the individual; yoga can be moderate exertion for some, aerobics class can be a vigorous activity for some, etc	Appropriate, understandable	Appropriate	Clear	Clear	Cutting wood, which he does regularly, is also a strenuous activity; the level of effort/intensity also depends on the individual
Wording of the questions	Items 4 and 5 are very confusing; had to read these repeatedly and did not grasp the 2 alternatives within each item	Overall clear, some items require reading twice (specifically 4 and 5)	Clear and make sense; needed to read some items twice, but able to relate to own physical activity	Somewhat confusing, needed to read repeatedly, especially those questions which include two options; but understandable after repeated reading	Initially confusing, needed to read through all questions to understand the logic; but then understandable and able to relate to own physical activity	Had to read through some questions repeatedly to be clear on differences in days/minutes between questions; overall understandable and able to relate to own physical activity
Response options	Appropriate, and for those items where the question wording is clear the answer options make sense	Make sense, although could also offer a 5-point rating	Make sense, but could also be formulated as 5-point rating	Clear and make sense	Appropriate	Appropriate

An overview of interviewees’ responses is provided in Table 1. Cognitive interviewing raised two issues which required minor revisions to the questionnaire:

#### Description and example images for intensity levels

With regard to the description and examples with images of physical activities according to intensity levels (light, moderate, vigorous), several participants made the point that – depending on the individual – example activities could also represent another intensity level. For example, an aerobics class could also constitute vigorous (rather than moderate) intensity for some people, and others might view strength training as a light (rather than moderate) activity. (Of note, this ambiguity is likely existent in other languages also.)

An addition of one further explanatory sentence was considered, to state that these are example activities for illustration, and that one activity may well be conducted at different intensity levels, but it was felt that this would detract from readability. Also, in verbalising their understanding, interviewees demonstrated that the meaning of the three intensity levels was in fact conveyed by the existing design.

**Fig. 1:**
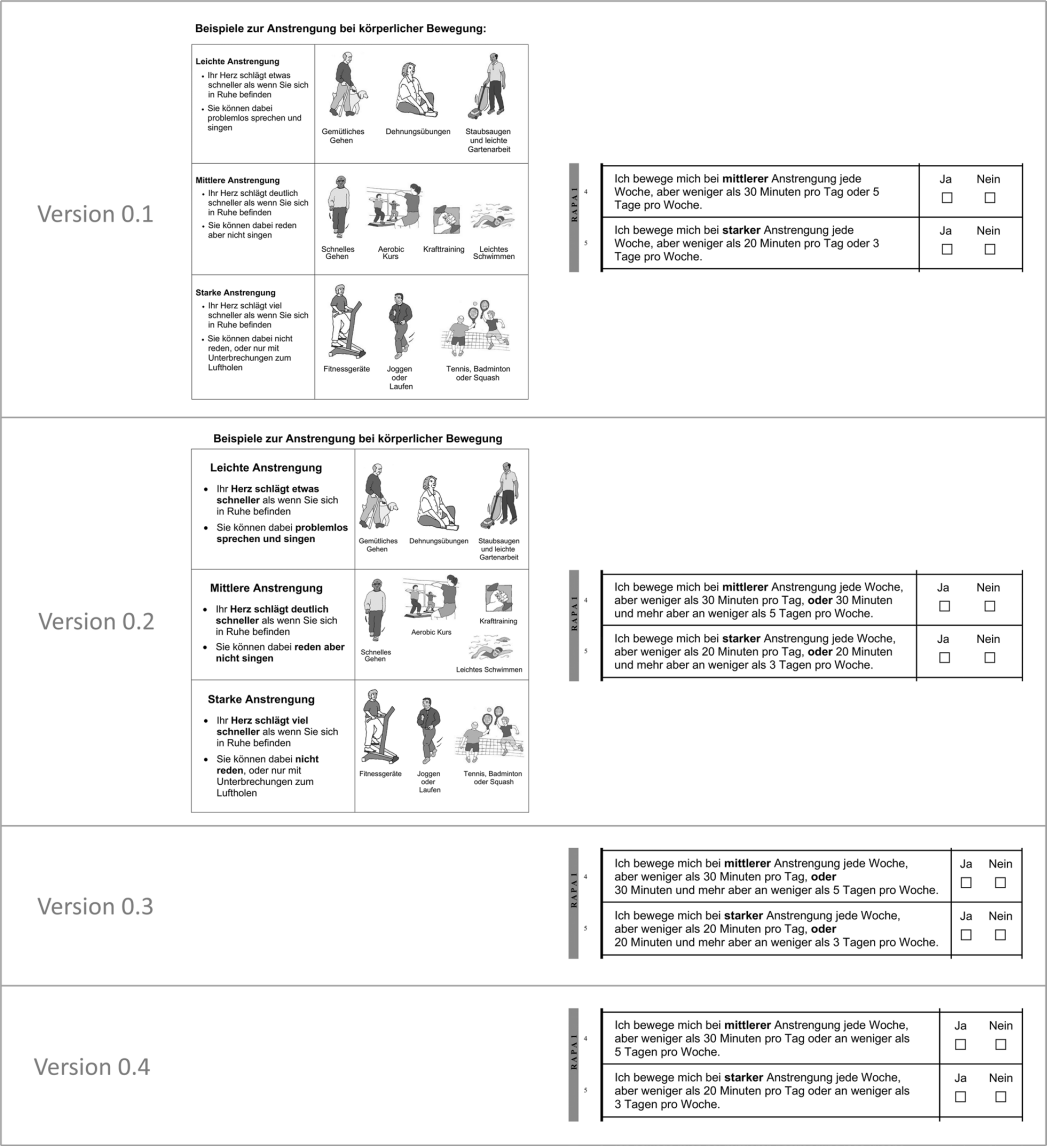
Iterative modifications to the translated Rapid Assessment of Physical Activity (RAPA) questionnaire during cognitive debriefing

One adjustment was made by increasing the font size and highlighting key words in the written description of intensity levels, to emphasise the discriminating physiological markers of heart rate and being able to talk/sing alongside the images of example activities (Fig. 1). Following this adjustment, the same point continued to be raised by several interviewees. However, when interviewees verbalised their thoughts in estimating their own intensity levels, it was apparent that they appropriately considered the physiological markers of intensity rather than categorising strictly by example activity. The adjustment in font size and key words was maintained, as it appeared to support the general readability and accessibility of the text.

#### Items 4 and 5

In the first block of interviews, the passages in RAPA items 4 and 5 ‘…every week, but less than 30/20 min a day or 5/3 days a week.’ caused some confusion and frustration with interviewees due to difficulty understanding. Verbalising their interpretations, interviewees stated that with regard to the statement ‘but less than 30/20 min a day’ they were clear on what they were being asked, but the passage ‘or 5/3 days a week.’ caused confusion.

Most interviewees interpreted this as being presented with two options: ‘I am being asked whether (A) I do moderate/vigorous physical activity every week but for less than 30/20 min each day; or (B) I do moderate/vigorous physical activity every week but less than 5/3 days a week.’ In this reading, there was confusion whether in (B) there is a minimum or maximum duration of minutes per day that applies. Because if someone does, e.g., 10 min of moderate physical activity for 4 days a week they could answer yes to (B), but they would answer no to (A) because they do not do it on every day of the week.

A first attempt was made in providing a more detailed wording for items 4 and 5: ‘I do moderate/vigorous physical activities every week, but less than 30/20 min a day, or 30/20 min or more on fewer than 5/3 days a week.’ The spacing of sentences was modified for greater clarity (Fig. 1). In five cognitive debriefing interviews, these modifications showed no improvement in terms of interviewees’ understanding.

A further attempt was made in reverting to the previous wording with minor amendment to the passage that caused confusion: ‘…but less than 30/20 min a day or on fewer than 5/3 days per week.’ This appeared to resolve the issue. In five cognitive debriefing interviews, interviewees stated that – although some of the questions (including items 4 and 5) required repeated reading to grasp their meaning – overall, the questionnaire made sense to them.

In the flow of sequentially answering items 1 to 7, which describe a stepwise increase in physical activity level, participants tended to complete items 1 to 3 swiftly, then hesitated to think about items 4 and 5, then often read ahead to items 6 and 7, and then tracked back to complete items 4 to 7 in sequence. This gives an indication that the meaning of items 4 and 5 was clarified by contrasting against the higher physical activity levels described in items 6 and 7.

## Discussion

Applying forward and back translation, a German version of the RAPA questionnaire has been produced for the Austrian context. Cognitive debriefing interviews with 13 adults aged 55 to 78 years have provided insights into respondents’ understandings and interpretations of questionnaire content, enabling further iterative refinement and thorough qualitative linguistic validation of the newly translated questionnaire.

Questionnaire-based assessment of physical activity constitutes a methodological cornerstone for physical activity research and promotion, and a considerable number of physical activity questionnaires have been developed in the past. The aforementioned scientific statement of the AHA identified 131 unique physical activity questionnaires originally developed for or feasible to deploy in healthcare settings [4]. A recent consensus statement by a panel of international experts across the fields of sports science, psychology and public health has affirmed the continued relevance of questionnaires for the assessment of physical activity [12]. This expert panel recommends identifying and translating already existing and published physical activity questionnaires (rather than developing new instruments) and highlights the importance of examining cultural and linguistic aspects of translated questionnaires through qualitative methods [12]. Qualitative data on cultural adaptation and linguistic validation unfortunately often remain unpublished or are afforded only brief descriptions in reports of psychometric validation studies. Similarly, qualitative investigations of respondents’ cognitive processes when completing a questionnaire feature less prominently in the literature, although these analyses can provide crucial insights for developers and users of questionnaires alike [13]. This article therefore intends to augment the literature by reporting on the process and outcome of the translation to German for Austria and qualitative validation of the RAPA.

Prominent examples for physical activity questionnaires which are available in the German language are the International Physical Activity Questionnaire (IPAQ) [14, 15], the European Health Interview Survey – Physical Activity Questionnaire (EHIS-PAQ) [16, 17] and the Global Physical Activity Questionnaire (GPAQ) [18, 19]. These questionnaires capture details of weekly amount of time spent sedentary (days, hours, and minutes) and/or doing certain activities (walking, cycling, physical work), allowing a judgement whether the person meets the recommendations for healthy levels of regular physical activity. In comparison to the RAPA questionnaire, these instruments offer more fine-grained and versatile data, including the option to derive weekly metabolic equivalents of task (METs), but at the expense of requiring respondents to recall relatively precise estimates of their physical activity behaviour. A qualitative study by Finger et al. [13] has demonstrated that such precise responses are more easily provided by employed and younger individuals who have a regular exercise or working schedule; but it can be problematic for other respondent groups to provide this information, in particular individuals aged 60 + years and those who do not work regularly (retirees, self-employed or unemployed) or those who performed physical activity irregularly [13]. This supports the design of the RAPA questionnaire with categorical response options that offer participants wide category brackets in terms of weekly frequency and duration of physical activity to compare themselves against. The present study demonstrates that even these comparatively simplified response categories require careful wording in order to achieve adequate comprehension and avoid confusion.

Respondents’ adequate understanding and estimation of levels of intensity of physical activity presents another potentially problematic aspect of physical activity questionnaires. This has been highlighted in several studies that qualitatively explored respondent’s thought processes when completing a questionnaire [13, 20, 21]. Problems that have been reported include: difficulty distinguishing between intensity levels; difficulty classifying activities when different intensity levels occurred within one activity; difficulty deciding which intensity category fits best (even for activities with homogeneous intensity level); not considering activities which do not cause sweating; and only thinking of activities which are mentioned as examples in the questionnaire text [13, 20, 21]. Of note, similar difficulties have been described for the self-reporting of sedentary behaviour (sitting activities) in physical activity questionnaires, for example distinguishing between reading a book in sitting versus reading while lying down [22]. These issues can lead to respondents making arbitrary choices, forgetting about relevant activities, and inappropriately excluding activities or including certain activities repeatedly. In this respect, the RAPA questionnaire differs from many physical activity questionnaires in that it offers respondents an introductory explanation of physical activity and intensity levels (light, moderate, vigorous) that is written in lay language, based on physiological markers of heart rate and respiratory rate and includes images of example activities for each intensity level. Participant feedback from the present study supports this presentation of intensity levels. In particular the point that one and the same activity may be conducted at different levels of intensity, or may constitute different intensity for different individuals, was brought up my most participants; but participants consistently demonstrated their appropriate understanding that they should be guided by heart rate and respiratory rate in classifying their own activities.

The RAPA questionnaire was developed with the intention to offer a brief standardised assessment tool for the clinical setting, to identify and monitor a patient’s physical activity level and initiate a conversation about physical activity promotion if indicated [3]. In Austria, as in many high-income countries, the promotion of regular healthy physical activity remains a high priority item on the public health agenda [11], with fewer than half of the Austrian population meeting WHO recommendations for regular aerobic physical activity, and only about a quarter meeting WHO recommendations for both aerobic physical activity and muscle strengthening exercise [23]. The ‘pandemic of physical inactivity’ [24] presents a major public health concern globally and particularly in high-income countries, where the prevalence of insufficient physical activity was reported at 36.8% (95% confidence interval 35.0–38.0) in 2016 and was observed to have increased over time [25]. In 2018, the WHO has defined the Global Action Plan on Physical Activity 2018–2030 which sets a target of a 15% relative reduction in the global prevalence of physical inactivity in adults and in adolescents by 2030 [26]. Proposed actions towards this target include strengthening the capabilities of healthcare professionals to assess and counsel patients on physical activity, and incorporating the promotion of physical activity across primary and secondary healthcare and social services [4, 26]. The successful implementation and scale-up of population physical activity interventions is undoubtedly determined by numerous implementation factors (barriers and facilitators) and often complicated by complex interplay between multiple factors [27, 28]. Nevertheless, studies of barriers and facilitators of physical activity promotion by healthcare professionals have consistently highlighted the importance of a suitable physical activity assessment tool that offers valid and reliable information and is practicable within health professionals’ limited time resources [29–31]. The new translation of the RAPA presented in this article may offer such a suitable assessment tool for the Austrian context.

### Limitations

Limitations to this study are acknowledged. The sample size was moderate, although the purposive sampling for gender, age and medical background achieved representation of relevant participant profiles, and this sample size is within the range of recommendations for pilot testing of newly translated instruments [32, 33]. Cognitive debriefing was able to uncover problems with clarity of the questionnaire’s wording and content through respondents’ verbalised reflections and through interviewers’ observations. It was not possible, however, to ascertain the accuracy of respondents’ self-ratings which would require a valid parallel assessment of physical activity such as a diary or a sensor measurement. It is acknowledged that the method in this study deviated somewhat from latest methodological guidance [7] in that a concept definition document was not created (however, a concept definition is in fact included in the RAPA preface); and the forward and back translators were not professional linguists but experienced clinicians and physical activity researchers who are proficient speakers of the source language and native speakers of the target language.

### Future research

In the next step, statistical psychometric validation of the new language version of the RAPA questionnaire is required to further support its content and construct validity by examining aspects such as convergent/concurrent and divergent/discriminant validity, including comparison of RAPA results with alternative valid measurements of physical activity.

## Conclusion

The process of translation and linguistic validation, including cognitive debriefing with 13 interviewees, has resulted in a consolidated version of the RAPA questionnaire in German for Austria that is appropriate for psychometric evaluation. In the future, the new translation of the RAPA may provide healthcare professionals in Austria with a suitable assessment tool for routine screening of patients’ physical activity levels, to raise awareness of physical activity recommendations and initiate physical activity counselling when indicated.

## Data Availability

The datasets used and/or analysed during the current study are available from the corresponding author on reasonable request.

## Abbreviations

EHIS-PAQ: European Health Interview Survey–Physical Activity Questionnaire
GPAQ: Global Physical Activity Questionnaire
IPAQ: International Physical Activity Questionnaire
METs: Metabolic Equivalents of Task
RAPA: Rapid Assessment of Physical Activity
WHO: World Health Organization
